# Quick assessment of hopelessness: a cross-sectional study

**DOI:** 10.1186/1477-7525-4-13

**Published:** 2006-03-01

**Authors:** Paul SF Yip, Yin Bun Cheung

**Affiliations:** 1 The Hong Kong Jockey Club Centre for Suicide Research and Prevention, The University of Hong Kong, Pokfulam Road, Hong Kong SAR; 2 MRC Tropical Epidemiology Group, London School of Hygiene & Tropical Medicine, UK

## Abstract

**Background:**

Lengthy questionnaires reduce data quality and impose a burden on respondents. Previous researchers proposed that a single item ("My future seems dark to me") and a 4-item component of the Beck's Hopelessness Scale (BHS) can summarise most of the information the BHS provides. There is no clear indication of what BHS cutoff values are useful in identifying people with suicide tendency.

**Methods:**

In a population-based study of Chinese people aged between 15 and 59 in Hong Kong, the Chinese version of the BHS and the Centre for Epidemiologic Studies – Depression scale were administered by trained interviewers and suicidal ideation and suicidal attempts were self-reported. Receiver operating characteristics curve analysis and regression analysis were used to compare the performance of the BHS and its components in identifying people with suicidality and depression. Smoothed level of suicidal tendency was assessed in relation to scores on the BHS and its component to identify thresholds.

**Results:**

It is found that the 4-item component and, to a lesser extent, the single item of the BHS perform in ways similar to the BHS. There are non-linear relationship between suicidality and scores on the BHS and the 4-item component; cutoff values identified accordingly have sensitivity and specificity of about 65%.

**Conclusion:**

The 4-item component is a useful alternative to the BHS. Shortening of psycho-social measurement scales should be considered in order to reduce burden on patients or respondents and to improve response rate.

## Background

Hopelessness is a system of negative expectations concerning oneself and one's future life [1]. It is an important concern in health and social care. Hopelessness is a strong predictor of suicide [2-4]. Suicide is closely associated with psychiatric illness, especially depression [5-7]. Though hopelessness is associated with depression, it is oriented to the future as opposed to the present state [8].

The Beck's Hopelessness Scale (BHS) was developed within the context of research on suicidal behaviour and depression [2,8,9]. It is a widely used instrument and has undergone numerous validation studies. A Chinese version has also been developed by Shek [10]. Useful though the BHS is, the length of 20 items is a discouraging factor. For an instrument to be useful in practical settings, it has to be short and easy to complete [11-13]. In particular, a psychological measure can be stressful for some respondents. It is desirable to reduce this stress by using shorter versions of the instrument [13]. Furthermore, long questionnaire may reduce data quality. A randomised experiment demonstrated that the length of quality-of-life questionnaires was inversely related to response rate [14,15]. Previous methodological studies suggested that the number of items in some scales can be reduced by about 70% without substantially compromising the measurement properties [16-18]. With these considerations, researchers should have a responsibility to use measurement scales that are as short as possible.

Based on confirmatory factor analysis, Aish, Wasserman and Renberg [19] commented that most of the BHS items measure a single factor, and that the number of items could be considerably reduced. They maintained that the following four items together predict the BHS scale almost perfectly:

Item 6: In the future I expect to succeed in what concerns me most.

Item 7: My future seems dark to me.

Item 9: I just don't get the breaks and there is no reason to believe I will in the future.

Item 15: I have great faith in the future.

According to Beck and Weissman [8], who maintained that the BHS measures 3 dimensions of hopelessness, items 6 and 15 measure an affective component, item 7 a cognitive component, and item 9 a motivational component of hopelessness. Aish, Wasserman and Renberg [19] further suggested that it might be possible to replace the 20-item BHS with item 7 (future seems dark) only, which "sum up all the essential aspects of hopelessness: no light at the end of the tunnel, the perception of a threatening uncertain future". In a study of patients with AIDS, Rosenfeld et al [20] found that a 3-factor model fitted the data better than a single factor model did. Nevertheless, they also found that the correlation between the 3 factors and suicidal ideation were very similar, the correlation coefficients ranged between 0.33 and 0.37. The correlation between the 3 factors and the Beck's Depression Inventory and the Hamilton Depression Rating Scale were also very similar (ranging between 0.53 and 0.61 and between 0.35 and 0.45, respectively). From the viewpoint of research on suicide and depression, there seems to be little to gain from attempting to differentiate the 3 factors.

Suicide is in a way an ultimate indicator of perceived poor quality of life. It has become one of the major threats to public health in Hong Kong, a predominantly Chinese society. In 2003, the suicide rate reached a historical high of 18.6 per 100,000 persons and was higher than the world average of 14.5 per 100,000 persons in 2000 [21]. The present study aims to shed light on the characteristics of the 20-item BHS and item 7 and the 4-item component of the BHS in relation to detecting suicidal ideation and suicidal attempt in the last 12 months and current depression as measured by the Center for Epidemiologic Studies – Depression Scale (CES-D) [22]. If the three BHS scores are associated with suicidality and depression in similar ways, it will testify to the comparability of the three scores and strengthen the case for advocating the shorter alternatives to the full version of BHS. This article does not aim to study suicidality and depression per se. A secondary aim is to examine the choice of cutoff scores on the BHS and its components to identify people as suicidal.

## Methods

### Survey design

This is a cross-sectional, community-based survey of the local resident population (between 15 and 59 years of age) of Hong Kong. All domestic helpers from overseas countries with conditional working visa are excluded from the targeted population. The sampling frame employed was based on the Frame of Quarters maintained by the Census and Statistics Department, which constitutes the most complete and up-to-date register of residential addresses in Hong Kong. A random sample of addresses was taken from the Frame. One subject was then randomly selected from each residential address. Informed written consent was obtained from the participant before the interview. Totally 2,219 persons aged between 15 and 59 participated in the study. The response rate was 62%. Given a sensitive topic like suicidal behaviour, this response rate was satisfactory as surveys on less sensitive topics in Hong Kong typically obtain a similar response rate [23]. Moreover, the demographic profile of this sample was found to be similar to those of the Hong Kong general population of that age range. Out of 2219 respondents, 70 had missing values in the BHS and 9 had missing values in the CES-D. Therefore the sample size for the present analysis was 2140.

### Measures

The survey began with a face-to-face interview, which included the Chinese version of the BHS (also known as C-HOPE) [10] and the CES-D, among other things. The respondents were then invited to self-complete a questionnaire that included questions on suicidal ideation and attempt in the past 12 months.

#### BHS

The Chinese version of the BHS was translated and validated in Hong Kong [10]. A slight modification in the design of the Chinese scale is that instead of asking the respondents to give a Yes-or-No answer, which was considered narrow in the response range, the respondents were asked to respond "Strongly Agree", "Moderately Agree", "Slightly Agree", "Slightly Disagree", "Moderately Disagree" or "Strongly Disagree" to the items [10]. The answers are coded as 1 to 6 in a way that a higher value represents a higher level of hopelessness. Scores on the Chinese BHS therefore ranges from 20 to 120. We extract the scores on item 7, which range from 1 to 6, and the sum of the scores on items 6, 7, 9 and 15, which range from 4 to 24, for comparison.

#### CES-D

The CES-D contains 20 items and the scores can range from 0 to 60. A Chinese version of the CES-D has been validated and used in Hong Kong [24,25].

#### Suicidality

The self-completed survey included a question "During the past 12 months, had you ever attempted to commit suicide?" and a question "During the past 12 months, had you ever considered suicide?" Respondents who gave positive replies to the first and second questions were considered to have suicidal attempt and suicidal ideation, respectively.

### Statistical analysis

We performed receiver operating characteristics (ROC) analysis using the scores on the BHS, the item 7, and the 4-item component to differentiate respondents with and without suicidal ideation and suicidal attempt in the last 12 months. A lack of discriminative power is indicated if the 95% confidence interval (CI) of the area under ROC curve (AUC) included the null value of 0.5; an AUC closer to 1 indicated a better discriminative power [26]. A non-parametric procedure was used to test the equality of the AUCs given by the correlated instruments [27]. The probability of reporting suicidal ideation and suicidal attempt was assessed in relation to the BHS scores by the locally weighted regression smooth method [28]. The relation between the CES-D and the BHS scores were assessed by ordinary least square regression. The R-square values of the models were compared for assessing the ability of the three scores in predicting CES-D scores. ANOVA was used to compare mean scores between groups of respondents. The statistical package STATA Version 8 (SataCorp, College Station, 2001) was used.

## Results and discussion

Table 1 shows the mean, standard deviation, median and observed minimum and maximum scores on the BHS, item 7, and the 4-item component of BHS. The agreement between mean and median suggest the absence of skewness. Almost the whole range of possible values was observed in each of them. Table 1 also shows the Pearson's correlation coefficient between the three scores. The BHS is strongly related to the scores on the 4-item component (r = 0.88) and item 7 (r = 0.71).

**Table 1:** Descriptive summary of and correlation between scores on the BHS and its components

						**Correlation**	
**Score**	**Mean**	**SD**	**Median**	**Min**	**Max**	**BHS**	**Item 7**
BHS	54.3	13.9	54	20	116		
Item 7 (Dark)	2.5	1.3	2	1	6	0.71	
Items 6, 7, 9, 15	10.1	3.6	10	4	24	0.88	0.80

One hundred and forty-three (6.7%; 95% CI 5.7 to 7.8%) respondents reported suicidal thoughts in the last 12 months; 38 (1.8%; 95% CI 1.3 to 2.4%) reported suicidal attempts. Table 2 shows the mean scores by suicidal ideation and attempt. Differences in mean values between the No-No, No-Yes, and Yes-Yes groups were evident for all three scores (each P < 0.01).

**Table 2:** Mean of scores on the BHS and its components by sucidality

	**Suicidal attempt**					
	**No**			**Yes**		
Suicidal ideation	BHS	Item 7 (Dark)	Items 6,7,9,15	BHS	Item 7 (Dark)	Items 6,7,9,15
No	53.5	2.4	9.9			
Yes	65.2	3.3	12.6	69.4	3.6	13.4

**Figure 1:**
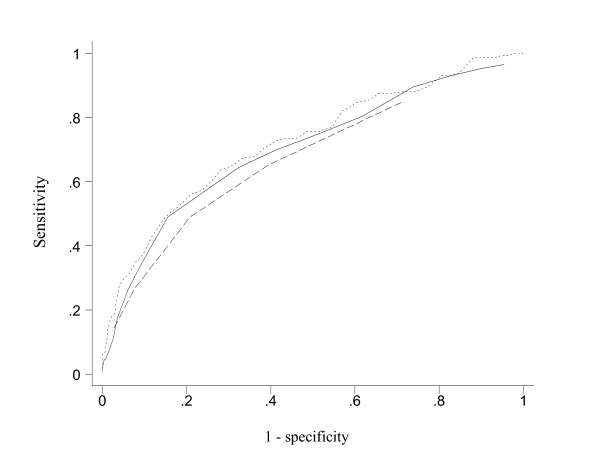
Receiver operating characteristics curves for predicting suicidal ideation in the last 12 months: dotted line for BHS (AUC = 0.72; 95% CI = 0.68 to 0.77), dashed line for item 7 (0.67; 0.62 to 0.72), and solid line for the 4-item component (0.70; 0.65 to 0.75) of BHS.

**Figure 2:**
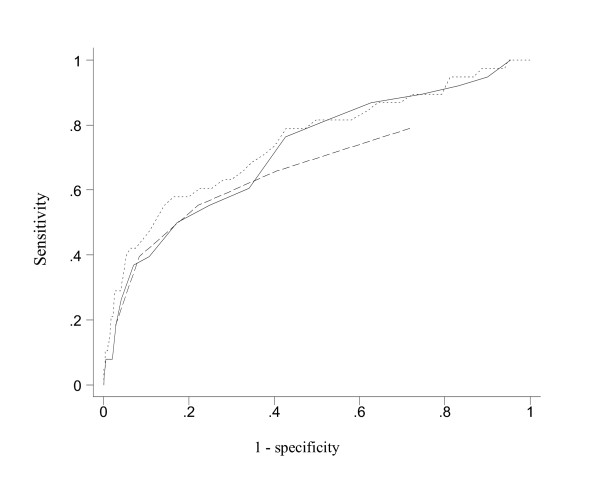
Receiver operating characteristics curves for predicting suicidal attempt in the last 12 months: dotted line for BHS (AUC = 0.75; 0.65 to 0.84), dashed line for item 7 (0.67; 0.57 to 0.78), and solid line for the 4-item component (0.72; 0.63 to 0.81) of BHS.

Figures 1 and 2 present the ROC curves for differentiating subjects with and without reporting suicidal ideation and attempt, respectively. While the ROC curves for item 7 were clearly inferior to those of the BHS, the 4-item sum gave ROC curves quite closely resemble those of the BHS, especially in the differentiation of people with and without suicidal ideation. The AUC's (95% CI) for item 7 (0.67; 0.62 to 0.72) was smaller than that for the BHS (0.72; 0.68 to 0.77) and the 4-item component (0.70; 0.65 to 0.75) in identifying people with suicidal ideation (P < 0.01 and P = 0.054, respectively). There was also a statistically significant difference in the ROC area for the 4-item component and the BHS (P = 0.042). A similar pattern was observed for differentiating people with and without suicidal attempts although the difference was not statistically significant (P > 0.05). The AUC's (95% CI) for BHS, the 4-item sum and the single item were, respectively, 0.75 (0.65 to 0.84), 0.72 (0.63 to 0.81) and 0.67 (0.57 to 0.78).

The cutoff points for the BHS that maximised the sum of sensitivity and specificity were 59 and 60 for suicidal ideation and attempt respectively. These cutoff values gave combinations of sensitivity and specificity of (67.1, 67.0) for suicidal ideation and (64.3, 67.6) for suicidal attempt. They corresponded to the position where the slope of the ROC curves was equal to one. The cutoff points for the 4-item BHS component were 11 for both suicidal idea and attempts, giving sensitivity and specificity of (65.8, 67.3) and (60.5, 65.9) respectively.

**Figure 3:**
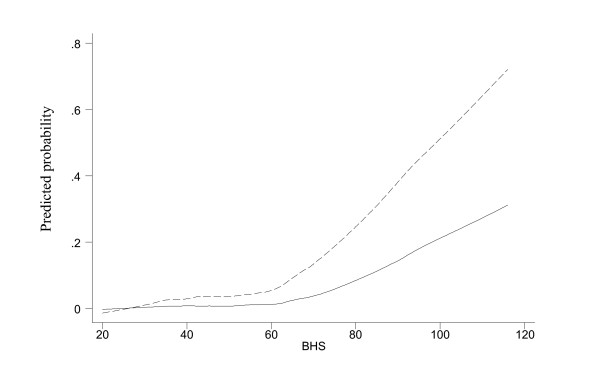
Smoothed probabilities of suicidal ideation (dashed line) and attempt (solid line) by BHS scores.

**Figure 4:**
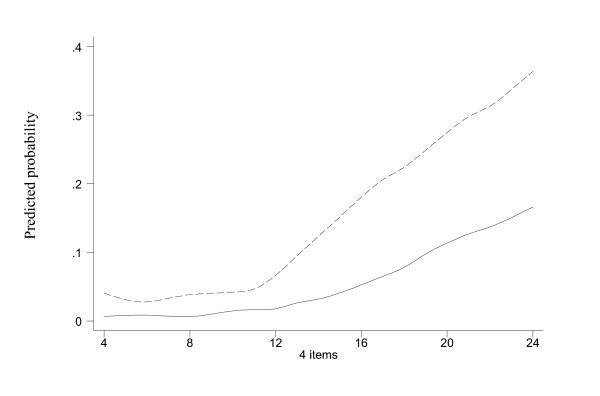
Smoothed probabilities of suicidal ideation (dashed line) and attempt (solid line) by sum of the 4 BHS item scores.

Figure 3 shows the smoothed risks of suicidal ideation and attempt according to the BHS. Figure 4 shows the risks according to the 4-item component. Both suggest that there is a clear threshold beyond which suicidality increased substantially. They corroborate with the ROC analysis in suggesting the cutoff points of about 60 for the BHS and 11 for the 4-item component. Thirty three percent of the respondents were above this BHS cutoff of 60 and 35% were above this 4-item cutoff of 11. We repeated the same analyses separately for respondents younger than 38 years old (median age in this sample) and at least 38 years old. The smoothed risks increased markedly at about 60 for the BHS and 11 for the 4-item component. So, as far as cutoffs are concerned, the two age groups are not different.

The mean (SD) of CES-D scores was 8.1 (9.6). Using least square regression, the BHS explained 26.3% of the variation in CES-D scores. Both item 7 and the 4-item BHS component explained 21.9% of the variation in CES-D.

Questionnaires should be short and quick to complete if they are to be clinically useful [12]. This is especially important if the questions can be emotionally stressful, as is the case of the screening for suicidal tendency and depression. Furthermore, studies seldom employ only one instrument. When there are multiple instruments in a survey, the questions can add up to impose a big burden on the respondents, especially those who are ill or distressed. Research on hopelessness and suicide were often conducted among patients with psychiatric or affective disorders [3,4]. We have drawn on data from a community-based study to elucidate how to utilise the BHS or its components to screen for people with suicidal tendency in the past 12 months.

Assessment of health and psychological constructs often requires composite measurement scales with high level of internal consistency. This implies that the items are correlated and therefore the information they provide overlap. Previous researchers have suggested that it might be possible to reduce the number of items in the BHS without substantial loss of information [19]. To our knowledge, however, it is the first time that this idea is assessed in relation to suicidality and its major determinant, depression. Our findings show that the 4-item component of the BHS was strongly correlated to the BHS score. Its ability to differentiate people with and without suicidality was similar to that of the BHS itself.

Furthermore, its ability to predict the CES-D score, which in turn is a strong determinant of suicide risk, was only slightly weaker than that of the BHS. When used alone, the item "My future seems dark to me" performed slightly inferior to the 4-item sum in detecting suicidality. There is a trade-off between discriminative power and length of questionnaires. One needs to balance the two aspects in a particular research context. Our findings suggest that a 4-item scale and the single item could be valuable alternatives to the full version of the BHS. Furthermore, our findings further strengthen the suggestion that many measurement scales can be shortened without losing substantial amount of information. Researchers should always consider such possibility before trying to impose lengthy questionnaires on patients and respondents.

The shape of a dose-response curve is critical in the formulation of screening criteria and intervention policy [29,30]. The relation between suicidality and BHS and the 4-item sum clearly expressed a threshold, at about score 60 and 11 respectively. Nevertheless, the sensitivity and specificity were not very high (~65%). Though a hopelessness measure can be useful in assisting the identification of people with suicidal tendency, additional means such as including other predictors in a simple questionnaire are likely to be required to have a more successful result in suicide prevention.

A limitation of the present study was that we did not actually administer item 7 or the 4 items of the BHS individually. Instead, the full version of the BHS was administered and the responses to the 20 items were used to obtain the scores on the single and 4 items. Another limitation is that the reliability of the three scores has not been examined. We have only compared them in a cross-sectional setting. Furthermore, suicidality was based on self-report. Further studies using other criteria to assess the relative performance of the BHS and its components, possibly using a randomised design to assign BHS or the 4 items only and using prospectively measured suicide risk, will be useful. Furthermore, we employed the Chinese version of the BHS. Similar analysis of the original BHS in English-speaking population will also be useful.

## Conclusion

The 4-item component is a useful alternative to the BHS. Shortening of psycho-social measurement scales should be considered in order to reduce burden on patients or respondents and to improve response rate.

## Competing interests

The author(s) declare that they have no competing interests.

## Authors' contributions

PY designed the survey and participated in the development the statistical framework, interpretation and discussion of the results. YBC conceived of the idea, developed the statistical framework and performed the analysis, participated in the interpretation and discussion of the results, and drafted the manuscript. Both authors read and approved the final manuscript.
